# Supplementary material for: Transverse tubule remodeling enhances Orai1-dependent Ca2+ entry in skeletal muscle
Source: eLife. 2019 Oct 28;8:e47576. doi: 10.7554/eLife.47576 (PMC6837846; doi:10.7554/eLife.47576)
Supplement: Transparent reporting form [file elife-47576-transrepform.docx]

***eLife’s*transparent reporting form**

We encourage authors to provide detailed information *within their submission* to facilitate the interpretation and replication of experiments.Authors can upload supporting documentation to indicate the use of appropriate reporting guidelines for health-related research (see [EQUATOR Network](http://www.equator-network.org/%20)), life science research (see the [BioSharing Information Resource](https://biosharing.org/)), or the [ARRIVE guidelines](http://www.plosbiology.org/article/info:doi/10.1371/journal.pbio.1000412) for reporting work involving animal research.Where applicable, authors should refer to any relevant reporting standards documents in this form.

If you have any questions, please consult our Journal Policies and/or contact us:[editorial@elifesciences.org](mailto:editorial@elifesciences.org).

**Sample-size estimation**

- You should state whether an appropriate sample size was computed when the study was being designed
- You should state the statistical method of sample size computation and any required assumptions
- If no explicit power analysis was used, you should describe how you decided what sample (replicate) size (number) to use

Please outline where this information can be found within the submission (e.g., sections or figure legends), or explain why this information doesn’t apply to your submission:

- When possible, sample sizes were estimated during study design.
- Sample sizes used for the electron microscopy analyses (Figs 1, 2, and S1) were based on our previously published studies (Boncompagni et al., 2017).
- Sample sizes for the single fiber (Figs. 3-5, 7, S2, and S4) and intact muscle contraction (Figs. 6, 7, S3, and S4) studies were determined from power analysis calculations (α=0.05; power=0.8) based on group mean and standard deviation values from both preliminary studies and our prior published works (Carrell, et al., 2016; Boncompagni et al., 2017).

**Replicates**

- You should report how often each experiment was performed
- You should include a definition of biological versus technical replication
- The data obtained should be provided and sufficient information should be provided to indicate the number of independent biological and/or technical replicates
- If you encountered any outliers, you should describe how these were handled
- Criteria for exclusion/inclusion of data should be clearly stated
- High-throughput sequence data should be uploaded before submission, with a private link for reviewers provided (these are available from both GEO and ArrayExpress)

Please outline where this information can be found within the submission (e.g., sections or figure legends), or explain why this information doesn’t apply to your submission:

- We performed each experiment in at least three or more animals.
- Technical replicates were defined as the number of fibers analyzed while biological replicates were defined as the number of animals analyzed (i.e. averaging all fibers obtained from a given animal).
- The number of replicates performed is clearly indicated in each figure.
- Averages ± SEM shown in the figures reflect technical replicates, although similar results were obtained when analyzing across biological replicates.
- When outliers were encountered, they were analyzed using the Chauvenet criterion to verify if an outlier is spurious.
- No high-throughput sequencing experiments were conducted in this study.

**Statistical reporting**

- Statistical analysis methods should be described and justified
- Raw data should be presented in figures whenever informative to do so (typically when N per group is less than 10)
- For each experiment, you should identify the statistical tests used, exact values of N, definitions of center, methods of multiple test correction, and dispersion and precision measures (e.g., mean, median, SD, SEM, confidence intervals; and, for the major substantive results, a measure of effect size (e.g., Pearson's r, Cohen's d)
- Report exact p-values wherever possible alongside the summary statistics and 95% confidence intervals. These should be reported for all key questions and not only when the p-value is less than 0.05.

Please outline where this information can be found within the submission (e.g., sections or figure legends), or explain why this information doesn’t apply to your submission:

- The final paragraph of the Materials and Methods section of the manuscript provides a detailed description and justification for the statistical analysis methods used in this study.
- Representative raw data and images are included in all figures with the exception of Fig. S1, (provide representative EM images for these studies could be provided during the revision process if needed).
- All data are presented as mean +/- SEM with n-values provided in figures.
- Statistical significance in Figs. 7 and S3, which involve comparisons across two groups, was determined using unpaired two-tailed Student’s test. For the all other results, a one-way ANOVA and *post-hoc* Tukey test was used determine significance across multiple comparisons. Histogram data shown in Figs. 3C and 4C were fit with a single Gaussian distribution.
- Results were considered statistically significance when p < 0.05. Exact p-values are not provided, but this could be updated during revision of the manuscript.

(For large datasets, or papers with a very large number of statistical tests, you may upload a single table file with tests, Ns, etc., with reference to sections in the manuscript.)

**Group allocation**

- Indicate how samples were allocated into experimental groups (in the case of clinical studies, please specify allocation to treatment method); if randomization was used, please also state if restricted randomization was applied
- Indicate if masking was used during group allocation, data collection and/or data analysis

Please outline where this information can be found within the submission (e.g., sections or figure legends), or explain why this information doesn’t apply to your submission:

- Four to six month old male WT mice were randomly assigned into one of four experimental groups: control group (non-exercised mice); <1 hour after exercise group (mice subjected to a 65’ treadmill running at increasing speeds and sacrificed within 1 hour from the end of the protocol); 6 hours after exercise group (mice subjected to a 65’ treadmill running at increasing speeds and sacrificed exactly 6 hours from the end of the protocol); 24 hours after exercise group (mice subjected to a 65’ treadmill running at increasing speeds and sacrificed exactly 24 hours from the end of the protocol).
- Masking was not used during group allocation, data collection and/or data analysis.

**Additional data files (“source data”)**

- We encourage you to upload relevant additional data files, such as numerical data that are represented as a graph in a figure, or as a summary table
- Where provided, these should be in the most useful format, and they can be uploaded as “Source data” files linked to a main figure or table
- Include model definition files including the full list of parameters used
- Include code used for data analysis (e.g., R, MatLab)
- Avoid stating that data files are “available upon request”

Please indicate the figures or tables for which source data files have been provided:

- A spreadsheet containing all numerical measurements used to generate each of the bar graphs and line plots presented in this study could be provided during the revision of the manuscript.
- This study did not generate large datasets (e.g. RNAseq, genomic, HTS data), code for data analysis programs, or utilize computational models.
